# Interfering in the ALK1 Pathway Results in Macrophage-Driven Outward Remodeling of Murine Vein Grafts

**DOI:** 10.3389/fcvm.2021.784980

**Published:** 2022-02-03

**Authors:** Alwin de Jong, Vincent Q. Sier, Hendrika A. B. Peters, Natalia K. M. Schilder, J. Wouter Jukema, Marie José T. H. Goumans, Paul H. A. Quax, Margreet R. de Vries

**Affiliations:** ^1^Department of Surgery, Leiden University Medical Center, Leiden, Netherlands; ^2^Einthoven Laboratory for Experimental Vascular Medicine, Leiden University Medical Center, Leiden, Netherlands; ^3^Department of Cardiology, Leiden University Medical Center, Leiden, Netherlands; ^4^Department of Cell and Chemical Biology, Leiden University Medical Center, Leiden, Netherlands

**Keywords:** vascular remodeling, ALK1 signaling, vein graft disease, ultrasound, macrophage

## Abstract

**Aims:**

Vein grafts are frequently used to bypass coronary artery occlusions. Unfortunately, vein graft disease (VGD) causes impaired patency rates. ALK1 mediates signaling by TGF-β *via* TGFβR2 or BMP9/10 *via* BMPR2, which is an important pathway in fibrotic, inflammatory, and angiogenic processes in vascular diseases. The role of the TGF-β pathway in VGD is previously reported, however, the contribution of ALK1 signaling is not known. Therefore, we investigated ALK1 signaling in VGD in a mouse model for vein graft disease using either genetic or pharmacological inhibition of the Alk1 signaling.

**Methods and Results:**

Male ALK1 heterozygous (ALK1^+/−^), control C57BL/6, as well as hypercholesterolemic ApoE3^*^Leiden mice, underwent vein graft surgery. Histologic analyses of ALK1^+/−^ vein grafts demonstrated increased outward remodeling and macrophage accumulation after 28 days. In hypercholesterolemic ApoE3^*^Leiden mice receiving weekly ALK1-Fc injections, ultrasound imaging showed 3-fold increased outward remodeling compared to controls treated with control-Fc, which was confirmed histologically. Moreover, ALK1-Fc treatment reduced collagen and smooth muscle cell accumulation, increased macrophages by 1.5-fold, and resulted in more plaque dissections. No difference was observed in intraplaque neovessel density. Flow cytometric analysis showed increased systemic levels of Ly6C^High^ monocytes in ALK1-Fc treated mice, supported by *in vitro* increased MCP-1 and IL-6 production of LPS-stimulated and ALK1-Fc-treated murine monocytes and macrophages.

**Conclusion:**

Reduced ALK1 signaling in VGD promotes outward remodeling, increases macrophage influx, and promotes an unstable plaque phenotype.

**Translational Perspective:**

Vein graft disease (VGD) severely hampers patency rates of vein grafts, necessitating research of key disease-driving pathways like TGF-β. The three-dimensional nature of VGD together with the multitude of disease driving factors ask for a comprehensive approach. Here, we combined *in vivo* ultrasound imaging, histological analyses, and conventional *in vitro* analyses, identifying the ambiguous role of reduced ALK1 signaling in vein graft disease. Reduced ALK1 signaling promotes outward remodeling, increases macrophage influx, and promotes an unstable plaque phenotype in murine vein grafts. Characterization of *in vivo* vascular remodeling over time is imperative to monitor VGD development and identify new therapies.

**Graphical Abstract:**
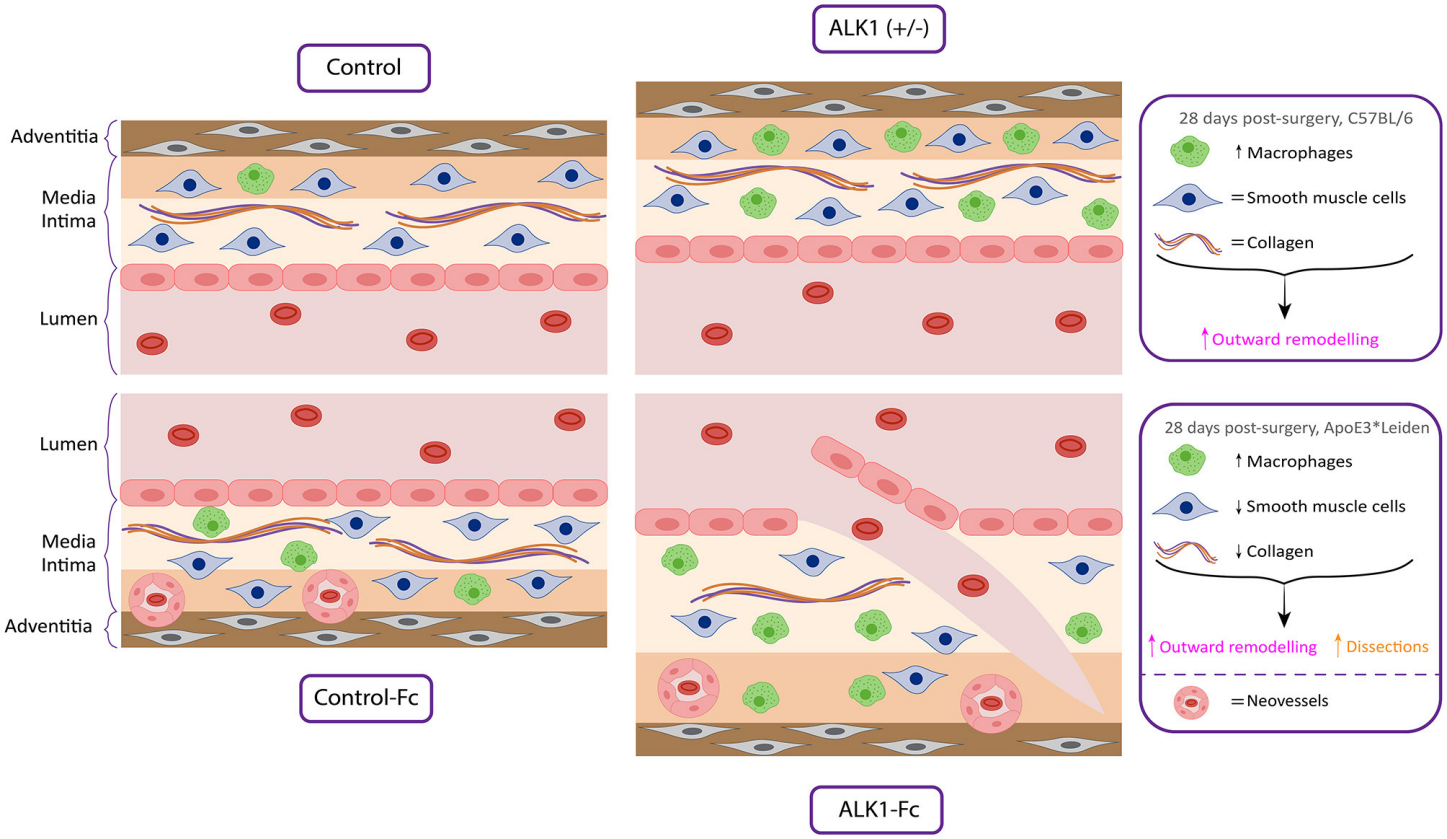
A reduction in ALK1 signaling induces outward remodeling, macrophage influx and promotes an unstable plaque phenotype in vein graft disease.

## Introduction

The vena saphena magna is the commonly used vein for bypass surgery in coronary or peripheral arterial circulation in patients with cardiovascular disease. After surgery, the vein graft requires arterialization to adapt to the pressure of the arterial circulation. Intimal hyperplasia develops through the proliferation and migration of vascular smooth muscle cells, ingrowth of neovessels, macrophage accumulation, and collagen deposition (1, 2). Critical processes for arterialization and successful long-term patency of the grafts are moderate intimal hyperplasia (IH) combined with outward remodeling (2). The beneficial process of proportional outward remodeling is often accompanied by moderate inflammation and angiogenesis (1, 3, 4). Excessive forms and overshooting of these mechanisms may induce disproportionate IH, causing inward, frequently partially occluding, vascular remodeling, and subsequent blood flow reduction. These processes together are indicated as vein graft disease (VGD). To reduce VGD, new therapeutic strategies are needed.

Members of the Transforming Growth Factor-β (TGF-β) superfamily are involved in the pathophysiology of VGD. Accordingly, TGF-β has been described as a driver for intimal hyperplasia, and fibrosis in vein grafts (5, 6). The TGF-β type I receptor activin receptor-like kinase (ALK)1 mediates signaling by TGF-β1 and TGF-β3 in the presence of TGFβR2 or high-affinity ligands BMP9 and BMP10 in the presence of BMPR2 that initiate downstream signaling *via* pSMADs. This is an important pathway in the fibrotic, inflammatory, and angiogenic processes in vascular disease (7–9). Considering that inflammation and angiogenesis play an important role in VGD, the ALK1 signaling pathway may be an interesting target in VGD. Neovessels facilitate both (i) the diapedesis of immune cells (10), and especially in the case of immature leaky neovessels, (ii) erythrocyte efflux, and subsequent intraplaque hemorrhage formation (11). Targeting ALK1 has been shown to reduce endothelial cell sprouting, impair neovessel ingrowth into Matrigel plugs, and decrease tumor angiogenesis (12, 13). Moreover, modulation of the ALK1 signaling pathway is suggested to regulate macrophage-mediated inflammatory processes and monocyte-macrophage differentiation (14).

In the current study, ALK1 heterozygous mice (ALK1^+/−^), as well as ALK1-Fc-treated hypercholesterolemic ApoE3^*^Leiden mice, were used to study the effects of interfering with ALK1 signaling *via* two approaches, a genetic and a pharmacologic approach. Homozygosity of ALK1 is embryonic lethal at mid-gestation due to severe vascular abnormalities associated with an increased expression of angiogenesis-related factors and reduced number of smooth muscle cells (8). With the background of ALK1 signaling in angiogenesis and inflammation, we aimed to elucidate the mechanism through which ALK1 affects vein graft remodeling. ALK1-Fc, consisting of the extracellular domain of ALK1 coupled to an Fc tail, acts as a scavenger receptor for BMP9 and BMP10. Specifically, ALK1-Fc sequesters BMP9/10, increasing their retention in the circulation and inhibiting BMP9/10-induced Smad-1 phosphorylation (pSMAD) and encompassing downstream transcription processes (15). Our study shows that in a mouse vein graft model (1), both ALK1 heterozygosity and ALK1-Fc ligand trapping were found to induce outward remodeling. Further investigations demonstrated that plaque angiogenesis was not affected by ALK1-Fc treatment, but an increased influx of inflammatory macrophages and a disbalance toward vessel wall destabilizing factors were observed.

## Materials and Methods

### Study Approval and Mice

This study was performed in compliance with the Dutch government guidelines and the Directive 2010/63/EU of the European Parliament. The Institutional Committee of the Leiden University Medical Centre approved all the animal experiments licensed under project numbers (11045, 116002016645). Written informed consent was obtained from the owners for the participation of their animals in this study. Heterozygous mice (ALK1^+/−^) and corresponding littermates were bred in our institute (16). Male C57BL/6 (ALK1^+/+^) and ALK1 heterozygous mice (ALK1^+/−^) were fed with a chow diet and ApoE3^*^Leiden (ApoE3^*^Leiden) mice were fed with a western type diet containing 1.0% cholesterol and 0.5% cholate (HFD) (Sniff Spezialdiäten, GMBH, Soest, Germany) (17). These mice were bred in our institute. In both breeding colonies, the mice were genotyped to confirm gene heterozygosity of *ALK1* or ApoE3^*^Leiden. Wild-type littermates were used as controls and all mice had free access to food and water.

### Experimental Design

Caval veins were obtained from male donor littermates with the same age (10–16 weeks) as the recipient mice. Both donor and recipient mice were anesthetized with an intraperitoneal (i.p.) injection consisting of a combination of midazolam (5 mg/kg, Roche), medetomidine (0.5 mg/kg, Orion), and fentanyl (0.05 mg/kg, Janssen). The depth of anesthesia was assessed by the pedal reflex. These caval veins were interpositioned in the carotid arteries of ALK1^+/+^, and ALK1^+/−^ mice, as previously described (18). After surgery and on the indication, buprenorphine (0.1 mg/kg, MSD Animal Health) was given as an analgesic. After 28 days, mice were anesthetized, pressure perfused with phosphate-buffered saline (PBS, Braun), and vein grafts were harvested, fixated in 3.7% formalin, and subsequently processed for paraffin embedding.

Pharmacological targeting of ALK1 signaling was performed in ApoE3^*^Leiden mice. These ApoE3^*^Leiden mice were fed with the HFD for 4 weeks before vein engraftment. Mice were randomized based on their plasma cholesterol values, measured enzymatically by using the Roche Diagnostic kit (Kit 1489437). Mice with plasma cholesterol values of >35 mM were excluded. Treatments consisted of 10 mg/kg bodyweight ALK1-Fc (RAP-041, an ALK1 extracellular domain/Fc fusion protein) or Fc control protein (12), which was administered via i.p. injection twice a week for 4 weeks in total.

### Histological and Immunohistochemical Analysis of Vein Grafts

Vein grafts were sectioned in sequential cross-sections of 5 μm thick made throughout the embedded vein grafts. The total vein was analyzed in equally spaced section. The plastic cuff was the starting point for mounting sections onto glass slides. HPS staining was performed using Hematoxylin, Phloxin 0.25%, and Saffron 0.3% to analyze the morphometrics of the vein grafts with ImageJ. The total vessel area and lumen area were measured. Vessel wall thickening was defined as the area between lumen and adventitia and determined by subtracting the lumen area from the total vessel area. The differences in the composition of the vein grafts were visualized by a Sirius red staining (Klinipath 80,115) to quantify the amount of collagen present. Anti-smooth muscle cell actin antibodies (1A4, 1:1,000, Dako) stained the vascular smooth muscle cells or anti-MAC3 (BD) (Clone M3/84, 550292 BD Biosciences) stained the macrophages, which were visualized by the DAB substrate complex. The images were loaded in ImageJ (FIJI), which calculated the percentage DAB isolated with the IHC toolbox FIJI plugin in the vein graft wall determined as a region of interest. The histological measurements were performed by a blinded experimenter. The macrophages were semi-quantitatively scored based on 0: none, 1: 10%, 2: 20–40% or 3: >40%.

### Ultrasound Measurements and Analyses

The animals were anesthetized with isoflurane and placed on the mouse imaging platform of the Vevo ‘3100' LAZR-X system (VisualSonics, FUJIFILM), where temperature, heart rate, and respiration rate were monitored in real-time. During the ultrasound acquisitions, anesthesia was maintained using a vaporized isoflurane gas system (1 L/min of oxygen 0.3 L/min air and 2.5% isoflurane). The concentration of isoflurane was adjusted accordingly to the pedal reflex and respiration rate to ensure adequate anesthesia. The region ranging from the salivary gland to the sternum was shaved and covered with ultrasound gel. Ultrasound imaging of vein grafts were performed with the MX550S transducer. Short-axis scanning 3D B-mode, and 3D color Doppler images were acquired weekly. Image visualization, reconstruction, and processing were realized with VevoLAB 3.2.6 software (FUJIFILM, VisualSonics). Both the cranial and caudal plastic cuff acted as landmarks to indicate the area for lumen and wall measurements. These lumen and wall measurements in 2D, as well as 3D, were performed by two persons, blinded to the grouping of mice in the two-study arms. Lumen and total vein graft area measurements were calculated using a software-corrected, freehand tracing of the region of interest on the short axis. The wall area was defined as the area in between the traced lumen and total vein graft area. Color doppler and EKV modes were used as supporting tools to clearly distinguish the vein graft from surrounding tissues (Supplementary Figure 1). Detailed analyses were performed using a 3D, short-axis ultrasound method with which lumen, wall, and outward remodeling area were calculated based on the mean of three measurement sites (caudal, medial, and cranial).

### Immunofluorescence

Neovessels were visualized by staining CD31 (1A4, sc376764 SantaCruz Biotechnology) with secondary goat anti-mouse Alexa Fluor 488 (A-11001, Thermo Fischer Scientific) and Hoechst 34580 1:1,000 (63493 Sigma-Aldrich). Images were obtained *via* laser scanning microscopy (LSM700, Zeiss). Polarization of macrophages was evaluated by staining CCR2 (CD192 Alexa luor 647, clone SA203G11, Biolegend) or CD206 (Alexa fluor 647, clone C068C2, Biolegend) in conjunction with CD107b (CD107b Alexa fluor 488, clone M3/84, Biolegend) into CD107b (Alexa Fluor 488, clone M3/84, Biolegend). The antibodies were diluted 1:100 in PBS supplemented with 1% normal goat serum and 1% BSA and incubated overnight at 4 degrees Celsius. The next day, the slides were washed, and incubated with Hoechst 34580 for 5 min. Prolong gold was used as a mounting medium. The integrated density of the CD107b, CCR2, and CD206 signal was quantified *via* a custom integrated density analysis script, by utilizing macro IJM (FIJI). Subsequent determination of double-positive cells (CD107b & CCR2 or CD107b & CD206) was performed by using a custom Python script run in Jupyter Notebook (Anaconda 3) and was represented as a percentage of the total CD107b positive cell count. The first percentile of the integrated density distribution was set as a threshold.

### Flow Cytometric Analysis

Flow cytometry was performed on peripheral blood mononuclear cells (PBMC) from ApoE3^*^Leiden mice treated with ALK1-Fc or control-Fc. Blood was obtained from the tail vein with EDTA as an anticoagulant. Erythrocytes were lysed in ACK lysis buffer (A1049201, Thermo Fischer) and washed twice with PBS supplemented with 0.1% heat-inactivated fetal bovine serum and 0.5 mM EDTA. Conjugated monoclonal antibodies to mouse CD11b PerCP Cy5.5 (M1/70, 1:100, BD Biosciences) and Ly6C APC (RB6-8C5, 1:250, BD Biosciences) were incubated for 20 min on ice. Flow cytometric acquisition was performed on a BD LSR II flow cytometer (BD Biosciences). Flow cytometric data were analyzed by using FlowJo V10.1 software (BD).

### Bone Marrow-Derived Cell Culture and ELISAs

In total, 250.000 murine bone marrow-derived monocytes were plated in 100 μl/well, and 200.000 M-CSF differentiated macrophages were plated in 500 μl/well. Both cell cultures were pre-treated with ALK1-Fc (0, 10, 100 ng/mL) or control-Fc (0, 10, 100 ng/mL) for 4 h and subsequently stimulated with 10 or 100 ng/ml lipopolysaccharide. After 24 h, the supernatant was stored at −20°C. The IL-6 and MCP-1 (BD Biosciences 550950, BD Biosciences 555260) concentrations were determined by ELISA according to the protocol (BD Biosciences) in the supernatant of these macrophages and monocytes.

#### mRNA Expression Analysis

Total RNA was isolated from paraffin-embedded vein grafts using the FFPE RNA isolation kit (Qiagen). Total RNA was quantitated using a NanoDrop 1,000 Spectrophotometer (Thermo Scientific). cDNA was synthesized using a High-Capacity cDNA Reverse Transcription Kit (Applied Biosystems) according to the manufacturer's protocol. qPCR was performed on ABI7500 Fast system using Taqman gene expression assays for Hprt1, TNFα, MCP-1, MMP2/9/14, TIMP1, BMP9, ID3, PAI, ELN, Col3A1, NLRP1/3, and IL-1β.

### Statistical Analysis

Differences in continuous variables between experimental groups were statistically assessed by using the unpaired parametric *T*-test in Graph Pad Prism 8 software. Data are represented as means ± SD unless stated otherwise. Significance was set at P < 0.05. Significant differences are graphically represented as ^*^P < 0.05, ^**^P < 0.01, and ^***^P < 0.001.

## Results

### Reduced ALK1 Signaling Shows an Increase in Outward Remodeling but Induces an Increase in Macrophage Presence

**Figure 1:**
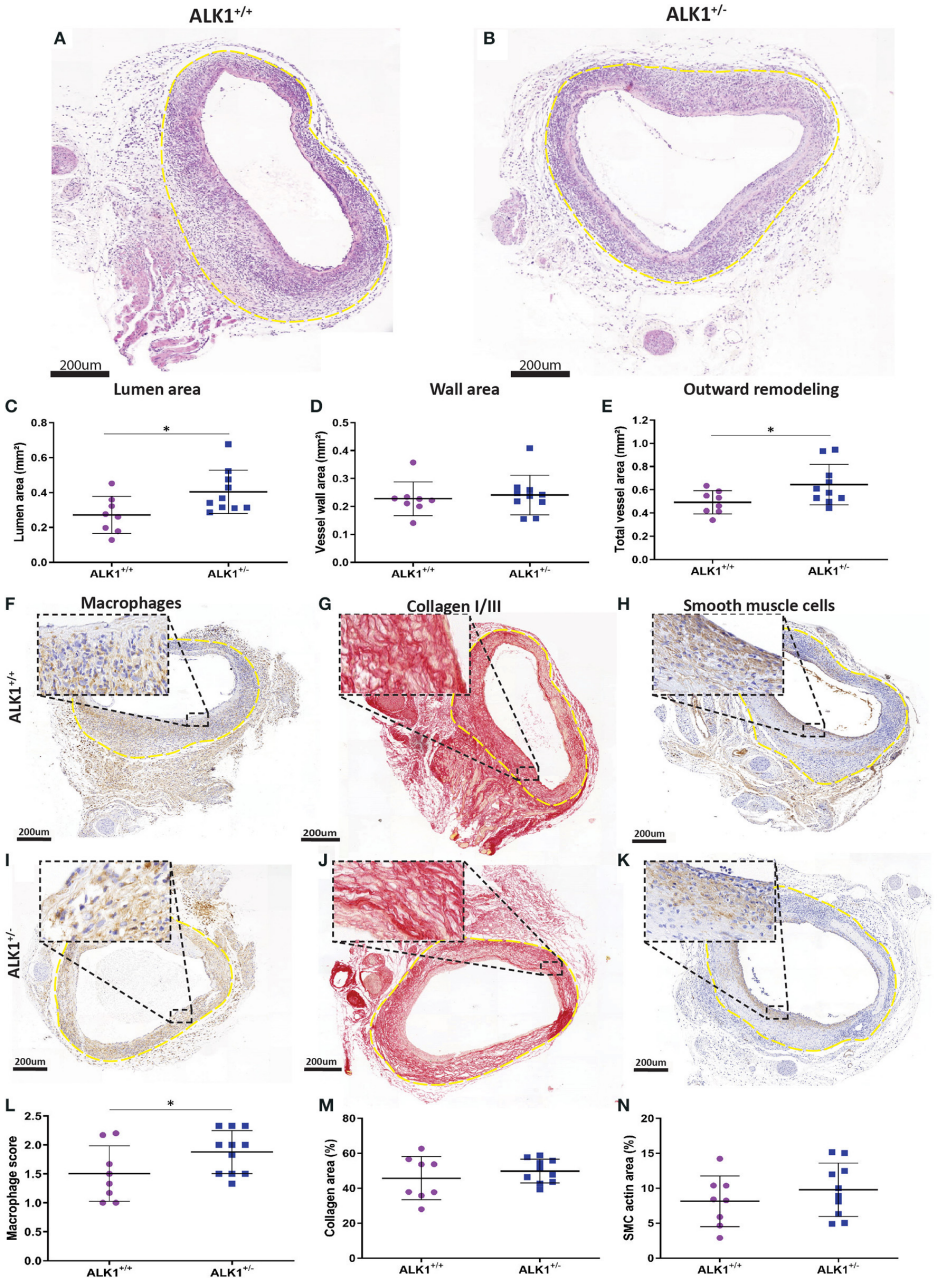
Morphometric and compositional analysis of ALK1^+/−^ and ALK1^+/+^ vein grafts. Vein grafts from ALK1 heterozygous mice (ALK1^+/−^, *n* = 7) were compared to vein grafts from ALK1^+/+^ (*n* = 10) mice. Typical examples of a vein graft from an ALK1^+/+^ mouse **(A)** and a vein graft from an ALK1^+/−^ mouse **(B)**. The outer adventitial layer is highlighted by the dotted yellow line. The morphometric parameters in mm^2^ represent the vein graft lumen area **(C)**, the vein graft wall area **(D)**, and the total vein graft area **(E)**. The composition of the vein grafts was evaluated by visualizing the macrophages **(F,I)** collagen **(G,J)** and the vascular smooth muscle cells **(H,K)** in vein grafts from ALK1^+/+^ and ALK1^+/−^ mice. Quantification of the macrophage score **(L)**, collagen area **(M)**, and smooth muscle cell positive area **(N)**. Statistical evaluation was performed with the unpaired parametric *T*-test, *p < 0.05.

To examine the contribution of the ALK1 signaling pathway to vein graft remodeling in mice, vein grafts from ALK1 heterozygous mice (ALK1^+/−^, *n* = 7) were compared to control mice (ALK1^+/+^, *n* = 10) mice since ALK1^−/−^ homozygosity is embryonic lethal (16) (Figures 1A,B). Notably, heterozygosity of ALK1 significantly increased vein graft lumen area by 38% (*p* = 0.03) (Figure 1C) and showed a 30% increased outward remodeling of the vein grafts (*p* = 0.04) (Figure 1E) while not affecting the vein graft wall area (Figure 1D). A significant increase in plaque macrophages was found in the ALK1^+/−^ mice in comparison to the ALK1^+/+^ animals (*p* = 0.03) (Figures 1F,I). In contrast, collagen (Figures 1G–M) and vascular smooth muscle cell content (Figures 1H–N), two factors that indicate stability, were unaffected by ALK1 heterozygosity. Thus, heterozygosity of ALK1 contributes to an increase in outward remodeling of the vein graft and induces an increase in macrophages present in the vein graft wall.

### ALK1-Fc Treatment of Vein Grafted ApoE3^*^Leiden Mice Results in Increased Outward Remodeling

**Figure 2:**
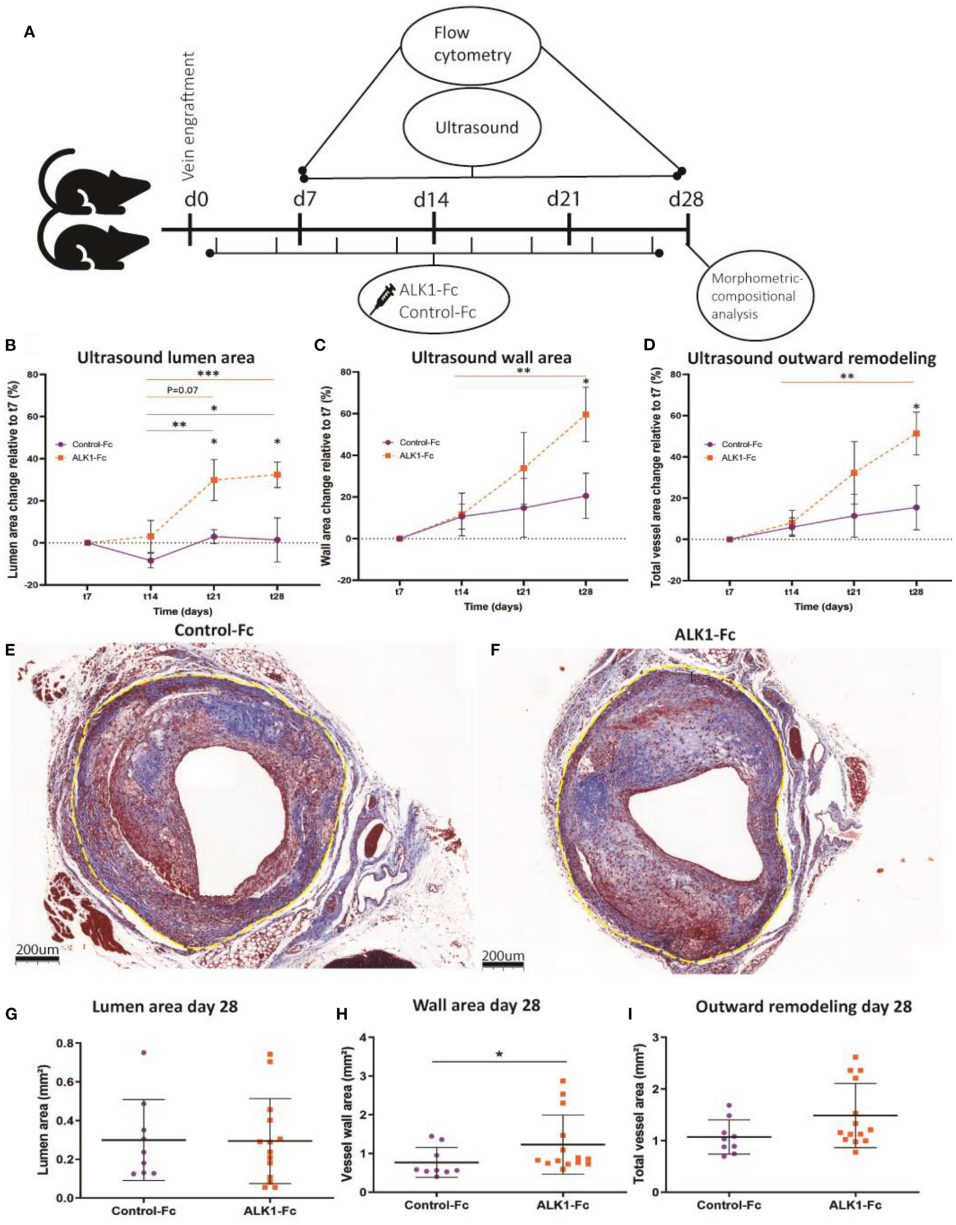
Non-invasive ultrasound analysis and histology analysis of control-Fc and Alk1-Fc treated vein grafts. High fat/cholesterol-fed ApoE3*Leiden mice were treated with ALK1-1Fc or control-Fc (10 mg/kg) twice a week for 28 days. **(A)** Ultrasound measurements were performed in vein grafted ApoE3*Leiden mice treated with control-Fc (*n* = 5) or ALK1-Fc (*n* = 6) at three separate locations in the vein graft, in which the vein graft lumen area **(B)**, vein graft vessel wall area **(C)**, and the total vein graft area **(D)** were quantified, represented as percentage change relative to day 7. For histology analysis, ALK1-Fc treated mice (*n* = 13) were compared to control-Fc treated mice (*n* = 9). Representative examples of a control-Fc treated vein graft **(E)**, and an ALK1-Fc treated vein graft **(F)** stained with Masson's trichrome. Lumen area **(G)**, wall area **(H)**, and outward remodeling **(I)** were measured 28 days after engraftment. Repeated measures were statistically tested by ANOVA and non-repeated measures by an unpaired *T*-test, and *p < 0.05, **p < 0.01, ***p < 0.001.

Ultrasound imaging provides real-time, quantitative anatomical and physiological information (19). To interfere with the ALK1 signaling pathway, hypercholesterolemic ApoE3^*^Leiden mice were treated with either ALK1-Fc or control-Fc and vein graft remodeling was quantified using both ultrasound imaging and endpoint histology (in two different cohorts) according to the set-up indicated in Figure 2A. Ultrasound imaging demonstrated that in the control-Fc treated group (*n* = 5) the lumen area only slightly differed in time with a small dip at the 14-day time point. Treatment with ALK1-Fc (*n* = 6) resulted in a gradual increase in lumen area from day 14 to a 10-fold non-significant increase at 21 days and resulting in a significant increase at 28 days compared to 7 and 14 days (*p* = 0.01, *p* = 0.004, Figure 2B). When comparing ALK1-Fc treatment to control-Fc at the individual time points, significant differences could be observed at the endpoint of 28 days (32 fold-increase by ALK1-Fc, *p* = 0.04) (Figure 2B). The vessel wall of both groups gradually thickened in time. In the control-Fc group, this resulted in a 1.9-fold increase between 14 and 28 days, although non-significant. Upon ALK1-Fc treatment, the observed gradual thickening was much stronger, demonstrated by a 5-fold increase in vessel wall growth at 28 days relative to 14 days after surgery (*p* = 0.04) (Figure 2C). This resulted in a 1.5-fold increased enlargement in size between the control-Fc and ALK1-Fc treated mice (*P* = 0.04, Figure 2C). Total vein graft area, which is a measure for outward remodeling, can be calculated based on adding lumen and vessel wall area together. In time, the vein grafts display an increase in outward remodeling. Although the control-Fc treated mice showed a small, non-significant increase in total vein graft growth, the vein grafts of the ALK1-Fc treated group demonstrated a steep incline in outward remodeling (6-fold increase at 28 days compared to 14 days, *p* = 0.02, Figure 2D). Comparing both groups, this resulted in a significant 3-fold increase upon ALK1-Fc treatment compared to control-Fc at 28 days post vein graft surgery (*p* = 0.03, Figure 2D). Histological analysis at 28 days (Figures 2E,F) revealed no differences in lumen area between both groups (Figure 2G). However, a significant 1.6-fold increase in vessel area can be observed (*p* = 0.03, Figure 2H), resulting in a non-significant (*p* = 0.08) trend toward outward remodeling (Figure 2I).

Taken together, ALK1-Fc treatment contributes to an increase in outward remodeling of vein grafts.

### Vein Graft Lesions Show a Destabilizing Phenotype Upon Treatment With ALK1-Fc

**Figure 3:**
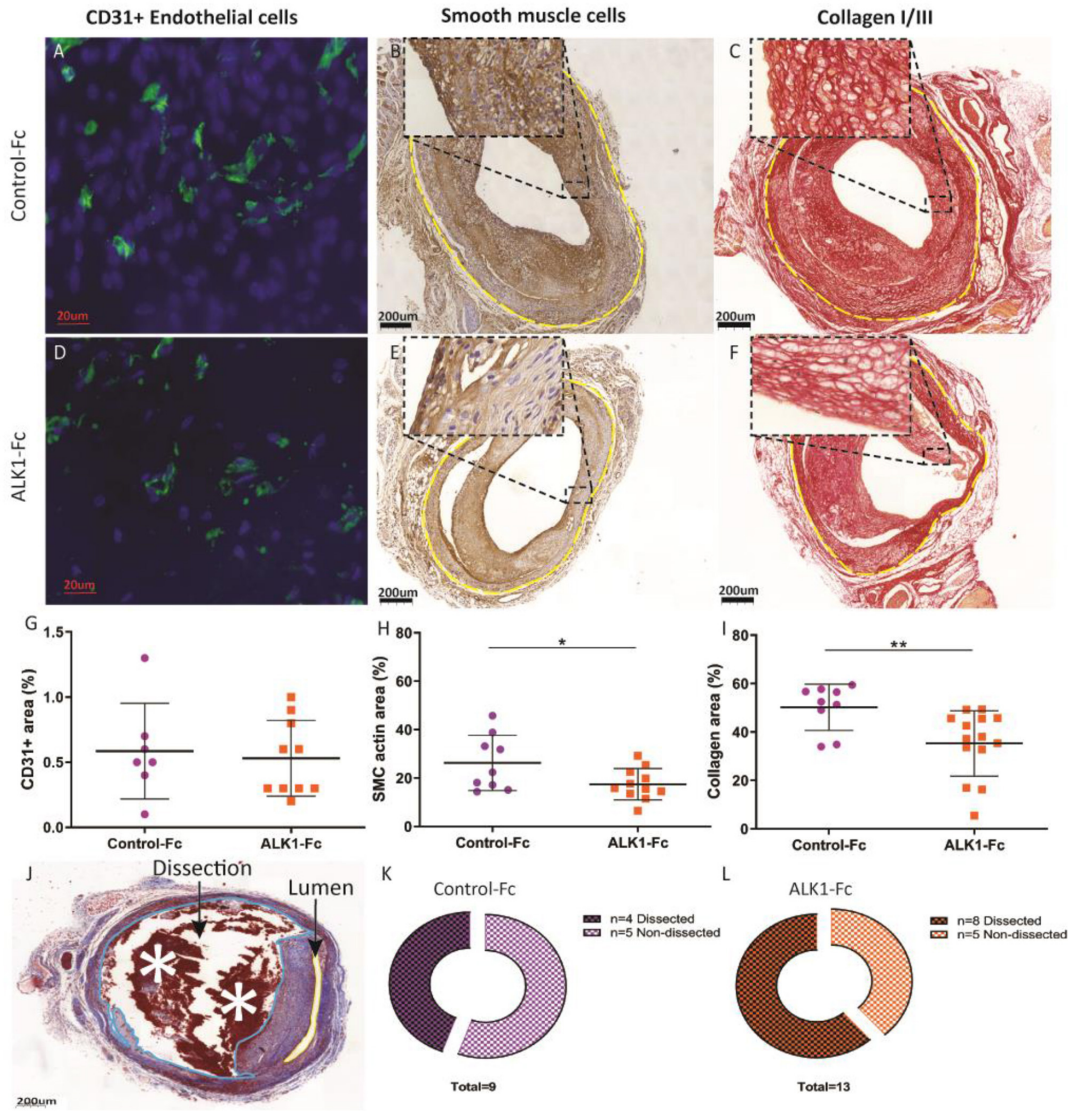
Compositional analysis of ALK1-Fc or control-Fc treated mice. ALK1-Fc treated mice (*n* = 11) were compared to control-Fc treated mice (*n* = 8). Representative examples of CD31 staining **(A)**, vascular smooth muscle cells **(B)**, and collagen **(C)** from a control-Fc treated mouse and examples of CD31 staining **(D)**, vascular smooth muscle cells **(E)**, and collagen **(F)** from an ALK1-Fc treated mouse. The vein graft is highlighted by the yellow dotted line. The relative presence of the CD31 staining, vascular smooth muscle cells, and collagen was identified in the vessel wall *via* calculation of a percentage area **(G–I)**. Destabilizing plaque dissections characterized by blood-filled gaps within the intimal hyperplasia reaching from the lumen to the adventitia **(J)**, dissections (dissection blue, lumen yellow) in control-Fc treated mice **(K)**, and ALK1-Fc treated mice **(L)**. Statistical evaluation was performed with the unpaired parametric *T*-test *p < 0.05, **p < 0.01.

Treatment with ALK1-Fc has been shown to reduce angiogenesis in murine tumor models (12). Since neovessels aggravate vein graft disease (11), the effect of ALK1-Fc treatment on vein graft angiogenesis was investigated. Neovessel density was not different between the two groups (Figures 3A,D,G), indicating that ALK1-Fc treatment did not affect angiogenesis in this vein graft model. Next, other lesion composition elements were investigated. Examination of smooth muscle actin expression showed that ALK1-Fc treatment reduced the percentage area of vascular smooth muscle cells significantly by 24% compared to controls (*p* = 0.04) (Figures 3B,E,H). In addition, the presence of collagen was significantly decreased by 30% in the ALK1-Fc treated group (*p* = 0.005) (Figures 3C,F,I). A phenomenon that is observed in unstable lesions is plaque dissections which are characterized by blood-filled gaps within the intimal hyperplasia, that reach from the lumen to the adventitia (20) (Figure 3J). The ALK1-Fc treated group showed an increase in plaque dissections compared to the control-Fc treated group (Figures 3J–L).

### Vein Graft Vulnerability Is Increased Due to the Influx of Pro-inflammatory Macrophages Upon ALK1-FC Treatment

**Figure 4:**
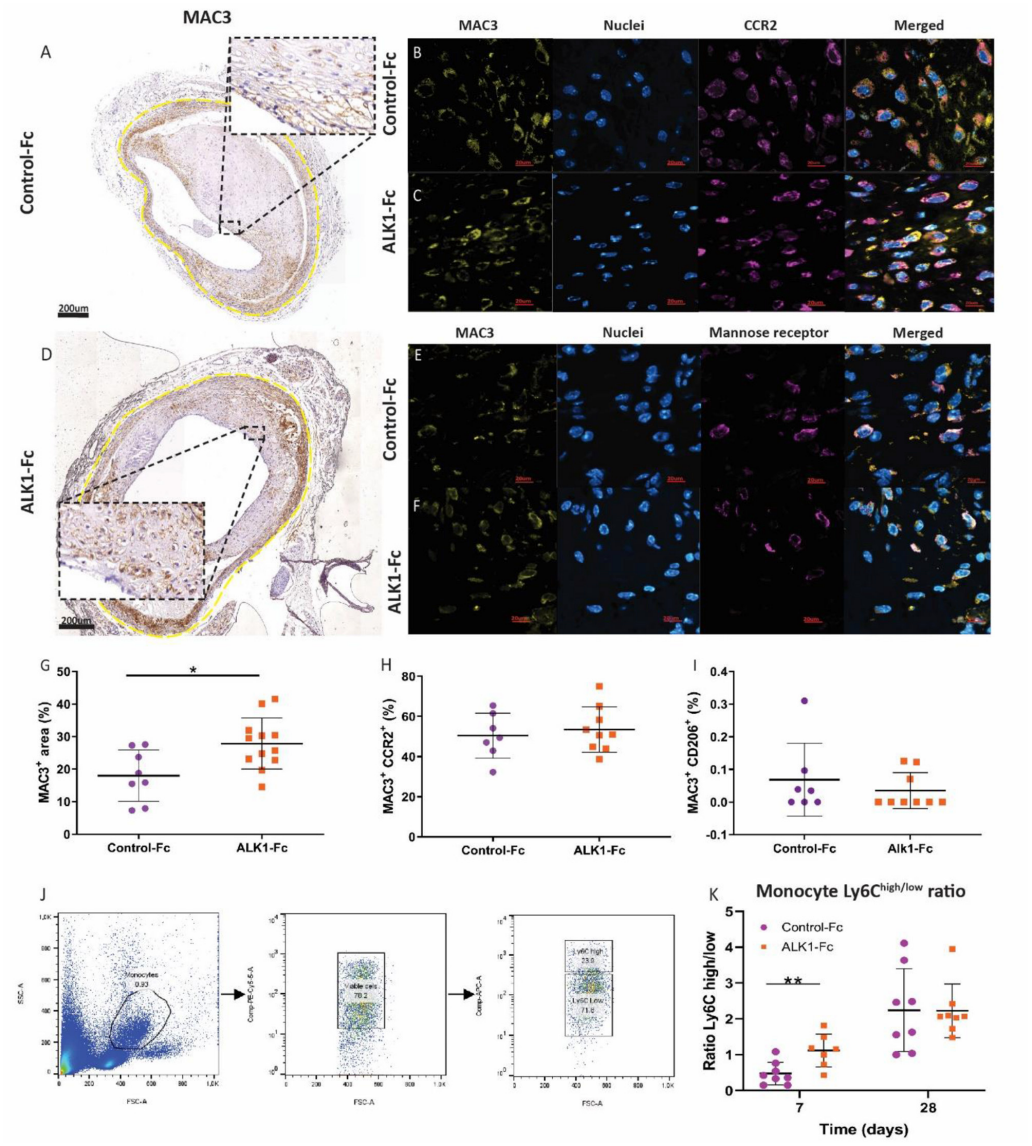
Macrophage content and polarization in vein grafts from ALK1-Fc or control-Fc treated mice. ALK1-Fc treated mice (*n* = 11) were compared to control-Fc treated mice (*n* = 8). Representative examples of a MAC3 (CD107b) **(A)**, MAC3, and CCR2 (CD192) from a control-Fc treated mouse **(B)** and an ALK1-Fc treated mouse **(C,D)**. MAC3 and the mannose receptor (CD206) from a control-Fc treated mouse **(E)** and an ALK1-Fc treated mouse **(F)**. The relative presence of the macrophages was identified in the vessel wall *via* calculation of a percentage area **(G)**. Double positive integrated densities of MAC3 and CCR2 positive macrophages **(H)** and MAC3 and mannose receptor-positive macrophages **(I)** areas were calculated. Monocytes in the blood that expressed Ly6C represented as a high/low ratio **(J,K)**. Statistical evaluation was performed with the unpaired parametric *T*-test, *p < 0.05, **p < 0.01.

Vein graft distension upregulates the expression of endothelial adhesion molecules. This triggers the influx of monocytes that subsequently differentiate into macrophages and promote vein graft disease (11). A 1.5-fold increase in macrophages was present in ALK1-Fc treated vein grafts as compared to control-Fc treated vein grafts (*p* = 0.001) (Figures 4A,D,G). Macrophages are key modulators and effector cells of the immune response. They may differentiate into pro-inflammatory, CCR2^+^ (CD192, formerly called M1) macrophages or anti-inflammatory mannose receptor^+^ (CD206, formerly called M2) macrophages. Both macrophage types were detected in the vein graft wall in both treatment groups, but no differences in the number of CCR2^+^ macrophages were observed between both treatment groups (Figures 4B,C,H). And also no differences in the mannose receptor^+^- macrophages (**Figures E,F,I**), indicating that ALK1-Fc treatment increased the total amount of macrophages in the vein grafts, but not in the specific subsets of CD192^+^ or CCR2^+^ macrophages. Regarding the presence of monocytes systemically in the blood, the ratio of Ly6C^high/low^ monocytes was significantly increased by 2.3-fold after the ALK1-Fc treatment compared to control-Fc treated mice at 7 days after engraftment (*p* = 0.008) (Figures 4J,K). However, no differences between the two groups of mice (Figure 2A) were observed in the Ly6C^high/low^ ratio after 28 days (Figures 4J,K), suggesting that ALK1-Fc induced an early systemic response in the pathogenesis of vein graft disease. In short, compositional analyses of murine vein grafts showed a decrease in stabilizing factors (collagen and smooth muscle cells) and an increase in destabilizing factors (monocytes and macrophages) and dissections upon treatment with ALK1-Fc.

### Monocytes and Macrophages Treated With ALK1-Fc Produce More Pro-inflammatory Factors

**Figure 5:**
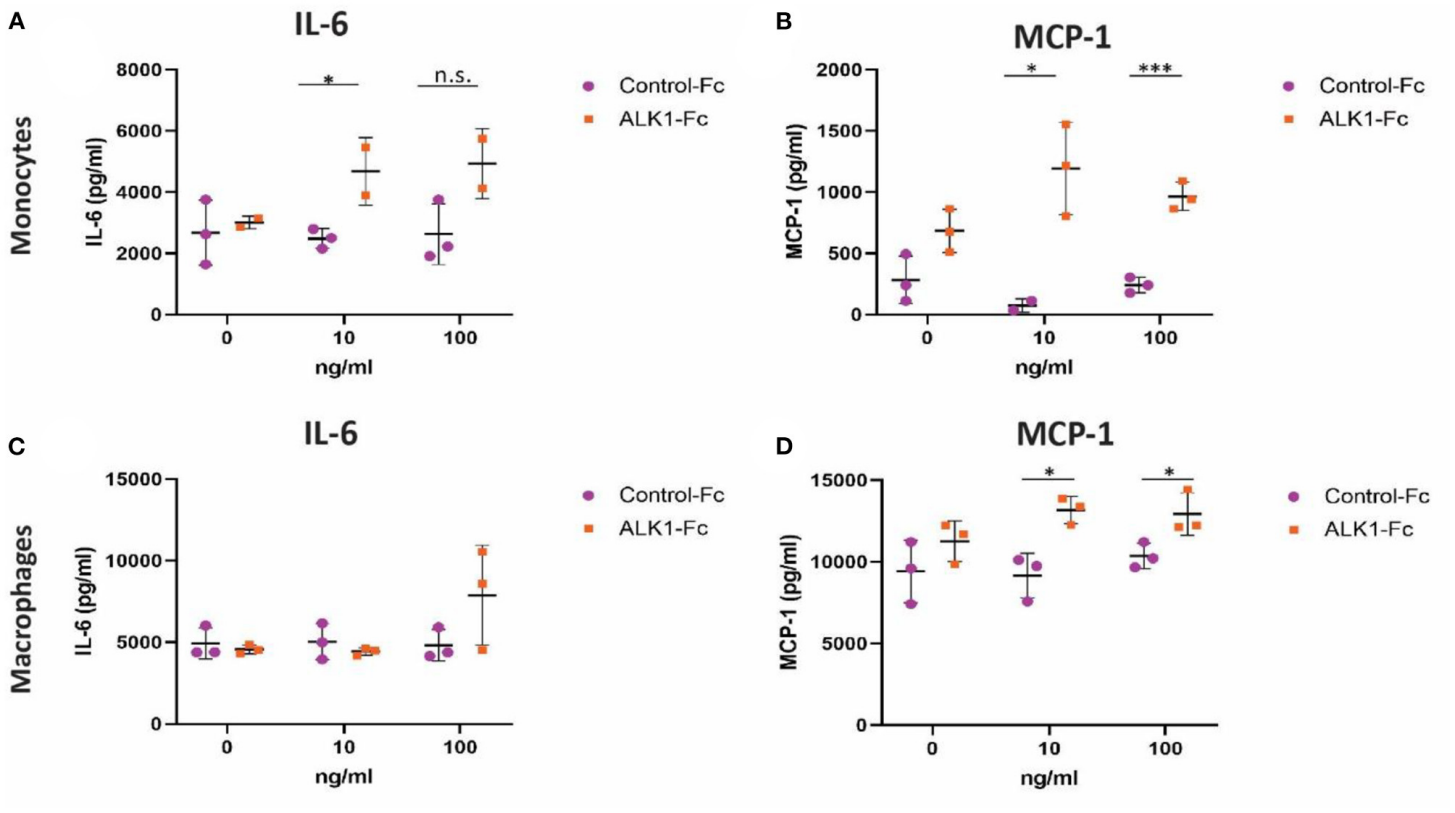
Production of pro-inflammatory cytokine IL-6 and chemokine MCP-1 by LPS-stimulated macrophages and monocytes upon treatment with ALK1-Fc or control-Fc. Monocytes (*n* = 3 experiments) and macrophages (*n* = 3) treated with 0, 10, or 100 ng/ml ALK1-Fc or control-Fc subsequently stimulated with 10 ng/ml LPS. The IL-6 **(A,C)** and MCP-1 **(B,D)** concentrations were determined in the supernatant. Statistical evaluation was performed with the unpaired parametric *T*-test, *p < 0.05, **p < 0.01, ***p < 0.001. n.s. = non-significant.

Since ALK1 heterozygosity and ALK1-Fc treatment had a profound effect on macrophages *in vivo*, we studied the effects of ALK1 on monocytes and macrophages in more detail, in particular, zooming in on pro-inflammatory cytokines. The production of IL-6 by monocytes *in vitro* was significantly increased by 1.8-fold in the 10 ng/mL ALK1-Fc treated monocytes compared to the control-Fc treated monocytes (Figure 5A). Furthermore, the production of MCP-1 by monocytes *in vitro* significantly increased by 16-fold 10 ng/mL ALK1-Fc (*p* = 0.03) and 4-fold after 100ng/mL ALK1-Fc treatment (*P* = 0.0007) (Figure 5B).

Although the concentrations of IL-6 released by macrophages were higher compared to monocytes, IL-6 release was not significantly affected by ALK1-Fc treatment in the macrophages (Figure 5C). However, the MCP-1 release by macrophages *in vitro* significantly increased by 1.5-fold 10 ng/mL ALK1-Fc (*p* = 0.01) and 1.2-fold after 100 ng/mL ALK1-Fc treatment (*P* = 0.04) (Figure 5D). These results indicate that ALK1-Fc treatment increases the inflammatory response in both monocytes and macrophages as was seen *in vivo*.

## Discussion

In the current study, we show that inhibition of ALK1 signaling either genetically or pharmaceutically impacts vein graft remodeling by inducing outward remodeling as well as increasing macrophage influx.

Histopathological studies and experimental vein graft models have shown that the inflammatory components of the innate immune system are present in all stages of vein graft adaptation (21). Crucial for a long-term good vein graft patency is the proper amount of vascular remodeling. On the one hand, the vein graft wall needs to thicken to cope with the arterial pressure resulting in inward remodeling, and on the other hand, the lumen should increase due to outward remodeling. A certain degree of inflammation is beneficial in the process of remodeling (22). Inflammatory cytokines and growth factors excreted by leukocytes invading the vessel wall contribute to the reendothelialization of the, during the surgery, damaged endothelium and activate smooth muscle cell migration and activation (1, 21, 22). This inflammatory trigger needs to dampen after the initial period to drive the remodeling process toward the positive outward remodeling. Our work has demonstrated that dampening the ALK1 signaling by half using the heterozygous ALK1^+/−^ mice induced outward remodeling toward the ideal situation without affecting the vein graft composition. A positive outward remodeling effect was also observed after ALK1-Fc treatment. When monitoring with *in vivo* ultrasound analysis this effect on outward remodeling seemed to be stronger than observed in histological sections, which might be due to tissue processing for histological analysis.

Pro-inflammatory cytokines modulate vein grafts (21), not only by stimulating the initial VSMC proliferation (23) but also by creating a positive loop of monocyte recruitment to the intima thereby increasing macrophage content in the vein graft wall (24). This is substantiated by our data showing the increased macrophage content in the Alk1^+/−^ mice. Moreover, upon treatment of cultured bone marrow-derived monocytes and macrophages with ALK1-Fc after a pro-inflammatory boost (stimulation with LPS), an increase in the production of the inflammatory chemokine MCP-1 is found. *In vivo*, upon ALK1-Fc treatment, positive outward remodeling was also accompanied by an increase in monocyte-mediated inflammation. This was also highlighted by the increase in Ly6C positive monocytes in the blood of ALK1-Fc treated mice.

It has already been shown that ALK1 plays important functions in EC and SMC regulation (25), as indicated by its expression during vasculogenesis and angiogenesis in early development (26). Although repressed in adult blood vessels, ALK1 is re-expressed upon processes of wound healing, tumorigenesis, and angiogenesis (27). ALK1-Fc treatment showed in a murine tumor model a reduction in tumor size and reduced angiogenesis. Vein grafts in mice on a non-atherogenic background such as the ALK1^+/+^ and ALK1^+/−^ mice do not display angiogenesis in thickened vein graft vessel walls. To study the effect of ALK1-Fc treatment on plaque angiogenesis we used ApoE3^*^Leiden mice that display larger lesions with lipid-rich cores and plaque angiogenesis in vein grafts (4). Our data showed no differences, however, in neovessels present in the vein graft wall. This can be explained that plaque angiogenesis is composed of heterogeneous processes and differs from tumor angiogenesis and therefore requires a different treatment strategy. This is what we demonstrated previously using VEGFR2 blocking antibodies that inhibit tumor angiogenesis that is unresponsive for plaque angiogenesis (28), and this could also be the case for ALK1-Fc. Alternatively, this unexpected lack of effect of ALK1-Fc treatment on intraplaque angiogenesis may be explained that ALK1 can be seen as a factor that mediates the maturation phase of angiogenesis, rather than the activation phase because *in vitro* transduction of endothelial cells with a constitutively active form of ALK1 leads to inhibition of proliferation, migration, and adhesion of these cells (29). But in the end, we can conclude that ALK1-Fc treatment with this concentration is not affecting intraplaque angiogenesis in vein graft disease.

There is some evidence for the involvement of ALK1 signaling in extracellular matrix (ECM) regulation (30). It has been described that the ratio of ALK1–ALK5 regulates ECM protein degradation *via* matrix metalloproteinases (31). Our data has shown that ALK1 heterozygosity did not affect the collagen content in the vein grafts, whereas the pharmacological approach by ALK1-Fc reduced the collagen content in the vein grafts, where it is not clear whether this is due to effects on collagen synthesis or turn-over. Furthermore, ALK1-Fc treatment reduced the number of VSMCs in the vein grafts, This is in agreement with the study of Seki et al., which showed overexpression of ALK1 increases proliferation of VSMCs and induced expression of alpha-smooth muscle actin (25).

Increasing evidence supports a role for ALK1 signaling in modulating monocyte differentiation *via* TGF-β binding to the TGF-βRII/ALK5 complex and exerting inhibitory signals for immune cells (7, 32). In the vein grafts of ALK1 heterozygous mice, an increased macrophage score was found as compared to wild-type mice. Moreover, similar macrophage activation and polarization toward a more inflammatory phenotype was found upon ALK1-Fc treatment. However, there is little information on the effects of ALK1 signaling in macrophages in general. A possible mechanism behind the observed macrophage accumulation upon ALK1 reduction and ensuing plaque destabilization upon ALK1-Fc treatment can be the antagonistic functions of ALK1 and ALK5. Upon ligand trapping with ALK1-FC, the Smad1/5/8 pathway is downregulated in macrophages. Subsequent upregulation of the ALK5 Smad2/3 pathway may result in macrophage activation and release of pro-inflammatory cytokines (33).

Vein grafts and macrophages are not likely to produce the ALK1 ligand BMP9, as underscored by two mechanisms. Firstly, ALK1-Fc acts directly *via* BMP9-ligand trapping, resulting in low BMP9-pSmad1 signaling and endoglin-dependent favoring of TGFβ-SMAD2 signaling in macrophages (34, 35). Secondly, the indirect sequestration of BMP9 by ALK1-Fc from the circulation reduces Smad1 production in the vein graft itself (36). These mechanisms are reflected by the increase in Ly6C^high^ monocytes in the blood of ALK1-Fc treated mice. Monocytes represent a systemic inflammatory response, while macrophages locally affect vein graft remodeling and inflammation, both of which are increased upon ALK1-Fc treatment. Reduced Smad1 signaling in ECs and VSMCs recruits macrophages to promote their pro-inflammatory status *via* the upregulation of ICAM and VCAM (12). In our experiments, an increase in macrophages present in the vein graft wall of both ALK1 heterozygous mice and ALK1-Fc treated mice was observed, suggesting that ALK1-Fc treatment exacerbates inflammation and masks the effect of ALK1-Fc on angiogenesis.

In conclusion, this study shows that inhibition of ALK1 signaling, either *via* a genetic approach or a pharmacological approach, in murine vein grafts promotes outward remodeling, increases macrophage influx, and promotes an unstable plaque phenotype, demonstrating a balancing role for ALK1 signaling in vein graft remodeling and disease.

## Data Availability Statement

The original contributions presented in the study are included in the article/Supplementary Materials, further inquiries can be directed to the corresponding author.

## Ethics Statement

The animal study was reviewed and approved by the Institutional Committee of the Leiden University Medical Centre (licensed under project numbers 11045 and 116002016645). Written informed consent was obtained from the owners for the participation of their animals in this study.

## Author Contributions

AJ, VS, NS, HP, and MV performed the experiments for the article. PQ and JJ provided funding. MG and MV did the first conceptualization. AJ, VS, MV, JJ, MG, and PQ wrote the manuscript. All authors contributed substantially to the discussion of content, reviewed, and edited the manuscript before submission.

## Funding

This work was supported by a research grant from the Leiden University Medical Center.

## Conflict of Interest

The authors declare that the research was conducted in the absence of any commercial or financial relationships that could be construed as a potential conflict of interest.

## Publisher's Note

All claims expressed in this article are solely those of the authors and do not necessarily represent those of their affiliated organizations, or those of the publisher, the editors and the reviewers. Any product that may be evaluated in this article, or claim that may be made by its manufacturer, is not guaranteed or endorsed by the publisher.

## Supplementary Material

Supplementary Figure 1 mRNA expression levels of genes involved in TGF-β/BMP signaling. Total RNA was isolated from ALK1-Fc treated ApoE3^*^Leiden mice (*n* = 6) and control-Fc treated ApoE3^*^Leiden mice (*n* = 6). The mRNA expression levels of genes involved in TGF-β/BMP signaling were assessed by qPCR. Statistical evaluation was performed with the unpaired parametric *T*-test, ^*^*p* < 0.05, ^**^*p* < 0.01.
