# Supplementary figures and images for: Interfering in the ALK1 Pathway Results in Macrophage-Driven Outward Remodeling of Murine Vein Grafts
Source: Front Cardiovasc Med. 2022 Feb 3;8:784980. doi: 10.3389/fcvm.2021.784980 (PMC8850982; doi:10.3389/fcvm.2021.784980)

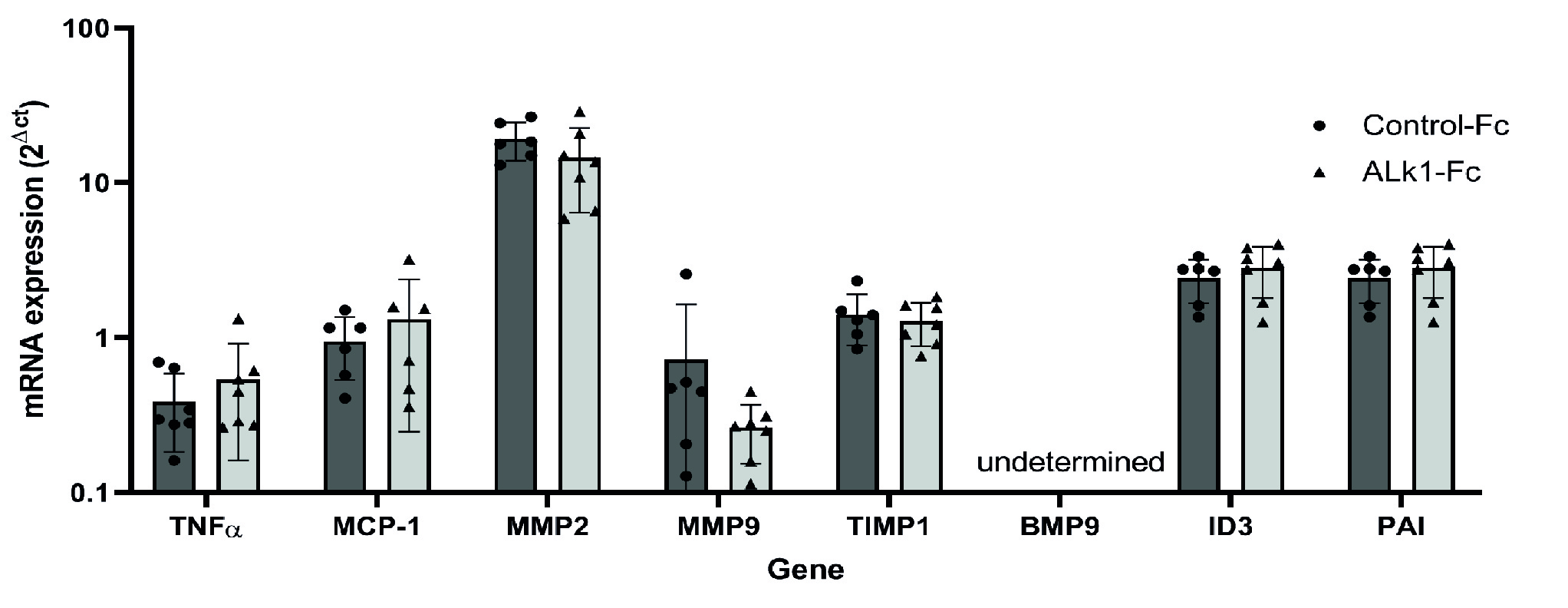

Supplement: Supplementary Figure 1 — mRNA expression levels of genes involved in TGF-β/BMP signaling. Total RNA was isolated from ALK1-Fc treated ApoE3*Leiden mice (n = 6) and control-Fc treated ApoE3*Leiden mice (n = 6). The mRNA expression levels of genes involved in TGF-β/BMP signaling were assessed by qPCR. Statistical evaluation was performed with the unpaired parametric T-test, *p < 0.05, **p < 0.01. [file Image_1.JPEG]
